# Effects of Obesity and Insulin on Tissue-Specific Recycling Between Cortisol and Cortisone in Men

**DOI:** 10.1210/clinem/dgaa896

**Published:** 2020-12-03

**Authors:** Anna J Anderson, Ruth Andrew, Natalie Z M Homer, Katherine A Hughes, Luke D Boyle, Mark Nixon, Fredrik Karpe, Roland H Stimson, Brian R Walker

**Affiliations:** 1 University/BHF Centre for Cardiovascular Science, Queen’s Medical Research Institute, University of Edinburgh, Edinburgh, UK; 2 Mass Spectrometry Core Laboratory, Edinburgh Clinical Research Facility, Queen’s Medical Research Institute, University of Edinburgh, Edinburgh, UK; 3 Oxford Centre for Diabetes, Endocrinology and Metabolism, University of Oxford, Churchill Hospital, University of Oxford, Headington, Oxford, UK; 4 Translational & Clinical Research Institute, Newcastle University, Newcastle upon Tyne, UK

**Keywords:** cortisol, cortisone, 11β-hydroxysteroid dehydrogenase 1, insulin, obesity

## Abstract

**Context:**

11β-Hydroxysteroid dehydrogenase 1 (11βHSD1) reduces inert cortisone into active cortisol but also catalyzes reverse dehydrogenase activity. Drivers of cortisol/cortisone equilibrium are unclear. With obesity, 11βHSD1 transcripts are more abundant in adipose, but the consequences for oxidation vs reduction remain unknown.

**Objective:**

This work aimed to determine whether 11βHSD1 equilibrium in metabolic tissues is regulated by insulin and obesity.

**Methods:**

A 2-phase, randomized, crossover, single-blinded study in a clinical research facility was conducted of 10 lean and obese healthy men. 11β-Reductase and 11β-dehydrogenase activities were measured during infusion of 9,11,12,12-[^2^H]_4_-cortisol and 1,2-[^2^H]_2_-cortisone, respectively, on 2 occasions: once during saline infusion and once during a hyperinsulinemic-euglycemic clamp. Arterialized and venous samples were obtained across forearm skeletal muscle and abdominal subcutaneous adipose. Steroids were quantified by liquid chromatography–tandem mass spectrometry and adipose tissue transcripts by quantitative polymerase chain reaction.

**Results:**

Neither whole-body nor tissue-specific rates of production of cortisol or cortisone differed between lean and obese men, however insulin attenuated the diurnal decrease. Whole-body 11β-HSD1 reductase activity tended to be higher in obesity (~ 10%) and was further increased by insulin. Across adipose tissue, 11β-reductase activity was detected in obese individuals only and increased in the presence of insulin (18.99 ± 9.62 vs placebo 11.68 ± 3.63 pmol/100 g/minute; *P* < .05). Across skeletal muscle, 11β-dehydrogenase activity was reduced by insulin in lean men only (2.55 ± 0.90 vs 4.50 ± 1.42 pmol/100 g/minute, *P* < .05).

**Conclusions:**

Regeneration of cortisol is upregulated by insulin in adipose tissue but not skeletal muscle. In obesity, the equilibrium between 11β-reductase and 11β-dehydrogenase activities likely promotes cortisol accumulation in adipose, which may lead to adverse metabolic consequences.

11β-Hydroxysteroid dehydrogenase type 1 (11βHSD1) is expressed in metabolically active tissues such as the liver, adipose tissue, and skeletal muscle. It catalyzes the 11β-reduction of cortisone to cortisol, thereby regenerating cortisol within these tissues (1). Clinical studies have shown increased 11βHSD1 expression and activity associated with type 2 diabetes (2) and in adipose tissue with obesity (3, 4). These observations, in combination with persuasive data from animal models (5-9), have led to inhibitors of 11βHSD1 being developed to prevent amplification of cortisol within target tissues for the treatment of type 2 diabetes (10). These have proven effective in phase 2 trials—lowering plasma glucose, blood pressure, and body weight—albeit not more so than existing therapies (11-13).

One reason for the limited efficacy of 11βHSD1 inhibitors in humans may relate to the “directionality” of 11βHSD1 activity, which has been studied to only a limited degree in obesity. Unlike 11β-hydroxysteroid dehydrogenase type 2 (11βHSD2) (14), which is an exclusive 11β-dehydrogenase that converts cortisol to cortisone and prevents inappropriate activation of mineralocorticoid receptors, 11β-HSD1 is a bidirectional enzyme in vitro (15). Moreover, Hughes and colleagues (16) and Dube et al (17) used stable isotope tracers with arteriovenous sampling to demonstrate the presence of bidirectional activity in vivo across both adipose tissue and skeletal muscle, reinforcing evidence from in vivo microdialysis in adipose tissue demonstrating both 11β-dehydrogenase and 11β-reductase activity (18). Appearance of cortisone in adipose (4) and skeletal muscle in these studies is far more likely to reflect dehydrogenase activity of 11βHSD1, rather than 11βHSD2, given the very low expression of the type 2 enzyme in these tissues (19, 20). It is possible that 11βHSD1 inhibitors could prevent both cortisol regeneration by 11β-reductase and inactivation by 11β-dehydrogenase in metabolically active tissues and that, similarly, the upregulation of 11β-HSD1 expression in adipose tissue in obesity may increase recycling between cortisol and cortisone, potentially being driven by hyperinsulinemia, with unpredictable effects on their overall equilibrium.

The redox balance of 11βHSD1 dehydrogenase and reductase activities is determined by the availability of NADPH, generated through the hexose-6-phosphate dehydrogenase (H6PDH) system. This has been elegantly demonstrated by altered glucocorticoid metabolism with genetic manipulation of *H6pdh* (21) in mice and also in disorders of glycogen storage (22). Insulin regulates 11βHSD1 expression and activity in various circumstances (23) and some of this effect may be attributed to shifting the nicotinamide adenine dinucleotide phosphate (NADP)/reduced NADP (NADPH) balance (24) to drive 11β-reductase activity and exaggerate glucocorticoid regeneration while simultaneously lowering glucocorticoid inactivation by 11β-dehydrogenase activity.

Against this background, we aimed to quantify bidirectional activity of 11βHSD1 in whole-body and across adipose tissue and skeletal muscle in lean and obese individuals using validated deuterated tracers (16, 25) and to distinguish the effects of hyperinsulinemia and obesity on 11β-reductase and 11β-dehydrogenase activities.

## Materials and Methods

The study protocol was approved by the local research ethics committee and written informed consent obtained. Participants were screened to exclude significant systemic illness or a history of diabetes mellitus or glucocorticoid use in the preceding 3 months. They were included if their alcohol intake was less than 21 units per week, their screening blood tests (full blood count, random blood glucose, kidney, liver, and thyroid function) were normal, and they were not receiving regular anticoagulation.

With 10 participants per group, the study was powered (> 90%) to detect a doubling in the rate of appearance of D3-cortisol or cortisone across adipose tissue (*P* < .05) based on variance measured in previous studies from our laboratory and given previous study results suggesting a 3-fold difference in gene expression and ex vivo enzyme activity as well as doubling of in vivo adipose 11βHSD1 activity in obesity (3, 26).

### Chemicals and reagents

Reagents were from Sigma or Steraloids unless otherwise stated. D4-Cortisol (9,11,12,12-[^2^H]_4_-cortisol) and D2-cortisone (1,2-[^2^H]_2_-cortisone) were from Cambridge Isotope Laboratories and ^133^Xenon from Nordion.

### Clinical protocol

Ten lean (body mass index 20-25 kg/m2) and 10 obese (body mass index > 30 kg/m2) healthy male volunteers were recruited to a randomized, 2-phase, crossover, single-blinded study, attending 2 weeks apart. All participants were between age 20 and 70 years. Participants attended at 8 am after an overnight fast at the clinical research facility and measurements were taken of clothed weight and height. Body fat was assessed by bioelectrical impedance (Omron BF-302).

**Figure 1.**
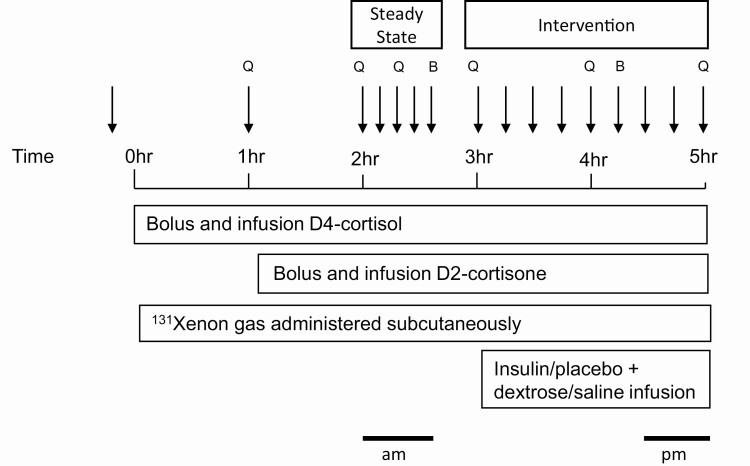
Schematic of clinical protocol. Arrows depict times of blood sampling and Q and B indicated where measures blood flow and needle biopsies were taken. Sampling in the steady-state phase was between the hours of 1100 and 1140 (ie, morning) and those samples used for analysis of the intervention phase between 1200 and 1400 (ie, afternoon).

The protocol is summarized in Fig. 1. The study visit commenced at 8 am and was completed 5 hours from the start of the tracer infusion. Participants continued to fast with only water to drink throughout the study visit. Participants remained supine throughout this period. Each participant attended 2 identical study visits except for a hyperinsulinemic infusion at one and placebo saline infusion at the other in random order.

A cannula (20-gauge) was inserted into the antecubital fossa for infusion of D4-cortisol and D2-cortisone. Three further retrograde cannulas were inserted to measure 11β-reductase and dehydrogenase activities across whole-body, forearm skeletal muscle, and abdominal subcutaneous adipose tissue: one cannula (20-gauge) was inserted into a vein on the dorsum of the hand that was placed in a heated box (controlled at 60 °C) for 5 minutes before sampling to arterialize the blood (oxygen saturation confirmed as > 98%); a second cannula (20-gauge) was placed in a branch of the cubital vein near the antecubital fossa on the opposite side (oxygen saturation confirmed < 40%), and an inflatable cuff was placed at the wrist and inflated (200 mm Hg, 2 minutes before sampling) to minimize contamination of venous blood from the hand; and a third cannula (18-gauge) was placed in a branch of the superficial epigastric vein in subcutaneous adipose tissue of the abdominal wall, inserted under the guidance of filtered red light (Schott UK Ltd) and the tip kept above the inguinal ligament to prevent contamination from venous drainage from the leg.

^133^Xenon (2 MBq) was injected into subcutaneous abdominal adipose tissue to measure adipose tissue blood flow (27). D4-Cortisol and D2-cortisone were administered diluted in 0.9% saline as an intravenous bolus of 1.4 mg and 76.0 µg followed by a continuous infusion of 0.7 mg/hour and 105.3 µg/hour, respectively; D2-cortisone was administered at 60 minutes because of its shorter half-life. Arterialized and venous blood were sampled before commencing the infusion, then at 60 minutes and again once steady state plasma concentrations were achieved after 2 hours (25). Blood was collected in lithium heparin tubes and plasma stored at –80 °C until analysis. At the time of sampling, blood flow through the skeletal forearm muscle and subcutaneous abdominal wall adipose tissue were measured using occlusion venous plethysmography (28) and washout of ^133^Xenon, respectively (29, 30). The “intervention” phase started at 3 hours. At the 2 visits, each participant received either a hyperinsulinemic euglycemic clamp or a placebo saline infusion in random order. For the clamp, insulin was infused at 35 mU/m^2^/minute based on body surface area (30). Capillary glucose concentrations were checked at 5-minute intervals (Accu-Chek, Roche) and dextrose (10%) infused at variable rates to maintain euglycemia (capillary glucose between 4.5 and 5.5 mmol/L). During the placebo phase, saline (0.9%) was administered in place of dextrose and capillary glucose measurements were taken every 5 minutes to provide blinding of participants. Arterialized and venous blood were sampled as stated earlier every 15 minutes for a further 120 minutes. Two subcutaneous abdominal adipose tissue biopsies were obtained by needle aspiration (31) at each visit. The first was performed at 160 minutes when tracers had reached steady state and a further biopsy at 285 minutes during the intervention period. These were immediately frozen and stored at –80 °C until analyses. After a total study time of 300 minutes, the participants were provided a meal and monitored to ensure they remained euglycemic before discharge.

### Laboratory analyses

Tracee and tracer steroids were quantified by liquid chromatography–tandem mass spectrometry with analysts blinded to randomization (32). Quantitative polymerase chain reaction was performed as described previously (33) in a subset of adipose biopsies, where sufficient quantities of paired samples were obtained (lean n = 7, obese n = 6). Intron-spanning primers were designed for use with probes within the Roche Universal Probe Library (UPL). Primer sequences and UPL probes numbers are as follows: *HSD11B1 forward: tctgtgttcttggcctcataga*; *reverse: gagctgcttgcatatggactatc*; *probe 8*; *HSD11B2 forward: gggggtcaaggtcagcat*; *reverse: cactgacccacgtttctcac*; *probe 64*; *PER1 forward: ctcttccacagctccctca*; *reverse: ctttggatcggcagtggt*; *probe 87*; *NR3C1 forward: ttttcttcaaaagagcagtgga*; *reverse: gcatgctgggcagttttt*; *probe 11*; *PPIA forward: atgctggacccaacacaat*; *reverse: tctttcactttgccaaacacc*; *probe 48* (33).

### Data and statistical analysis

Rates of appearance (Ra) of cortisol, cortisone, and 9,12,12-[^2^H]_3_-cortisol (D3-cortisol) were calculated as reported previously (16). Data were analyzed using Statistica (Dell) and reported as mean ± SEM unless otherwise stated. Absolute concentrations of steroids in blood were compared by repeated-measures analysis of variance (ANOVA) with Fisher least significant difference post hoc test, using all time points. Individual missing data points from blood sampling were imputed by taking the mean of the immediately preceding and subsequent values for repeated-measure analysis. Steady-state data for individual participants were expressed as mean between time 120 to 180 minutes. Differences between groups and phases (influence of visit, obesity, circadian timing, and hyperinsulinemia on glucocorticoid metabolism) were assessed by 2-way or repeated-measures ANOVA, with Fisher post hoc tests as described in the figure and table legends. Generation across adipose and skeletal muscle was assessed by single-sample *t* test compared with zero. Data across adipose were incomplete because of failure in sampling from the intra-adipose cannula in the abdominal wall in some individuals, thus analysis for the intervention phase across adipose was restricted to 270 to 300 minutes where data were available for n = 9 participants, except in the lean group during the insulin phase (n = 8).

## Results

### Participant characteristics

Ten lean and 10 obese healthy men (Table 1) matched for age attended 2 study visits. Glucose disposal rates did not differ between lean and obese participants.

**Table 1.** Participant characteristics

No.	Lean	Obese	*P*
	10	10	
Age, y	50.0 ± 10.4	50.5 ± 10.4	.97
Weight, kg	72.25 ± 4.89	101.13 ± 11.03	< .05
Height, m	1.74 ± 0.07	1.75 ± 0.07	.80
BMI, kg/m^2^	23.77 ± 1.20	32.92 ± 2.72	< .05
Body fat, %	20.54 ± 6.14	29.88 ± 4.45	< .05
WHR	0.99 ± 0.05	1.0 ± 0.05	.74
Fasting glucose, mmol/L	5.1 ± 0.4	5.3 ± 0.4	.15
Glucose disposal rate/M value mg/min/kg	10.3 ± 6.7	12.6 ± 3.9	.39
Total cholesterol, mmol/L	4.3 ± 0.4	4.7 ± 0.9	.41
HDL cholesterol, mmol/L	0.9 ± 0.3	1.0 ± 0.2	.32
LDL cholesterol, mmol/L	2.6 ± 0.5	2.9 ± 0.8	.36
Triglycerides, mmol/L	2.1 ± 1.5	1.4 ± 0.5	.08
Systolic BP, mm Hg	142.2 ± 23.6	141.0 ± 14.8	.86
Diastolic BP, mm Hg	81.6 ± 11.7	86.2 ± 11.5	.36

Data are mean ± SD, compared by *t* test.

Abbreviations: BMI, body mass index; BP, blood pressure; HDL, high-density lipoprotein; LDL, low-density lipoprotein; WHR, waist-to-hip ratio.

#### Whole-body glucocorticoid kinetics

**Figure 2.**
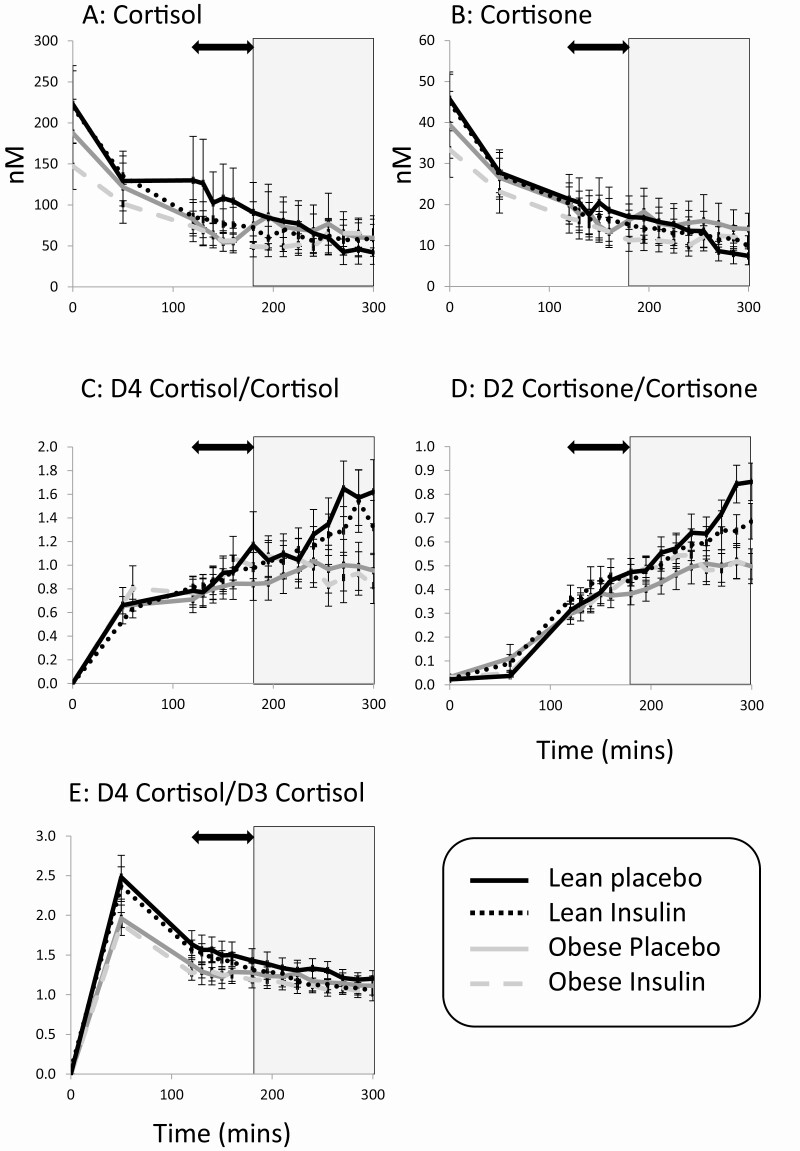
Circulating concentrations of A, cortisol; B, cortisone and tracer:tracee ratios; C, D4-cortisol/cortisol; D, D2-cortisone/cortisone; and E, D4-cortisol/D3-cortisol during tracer infusion. Data are mean ± SEM, n = 9-10/group. Black arrow represents steady-state period (120-180 minutes) and shaded area represents period of insulin/placebo intervention. Lean = black, Obese = gray; placebo intervention = solid, insulin intervention = dotted. Data were compared by repeated-measure analysis of variance with Fisher least significant difference post hoc tests.

**Figure 3.**
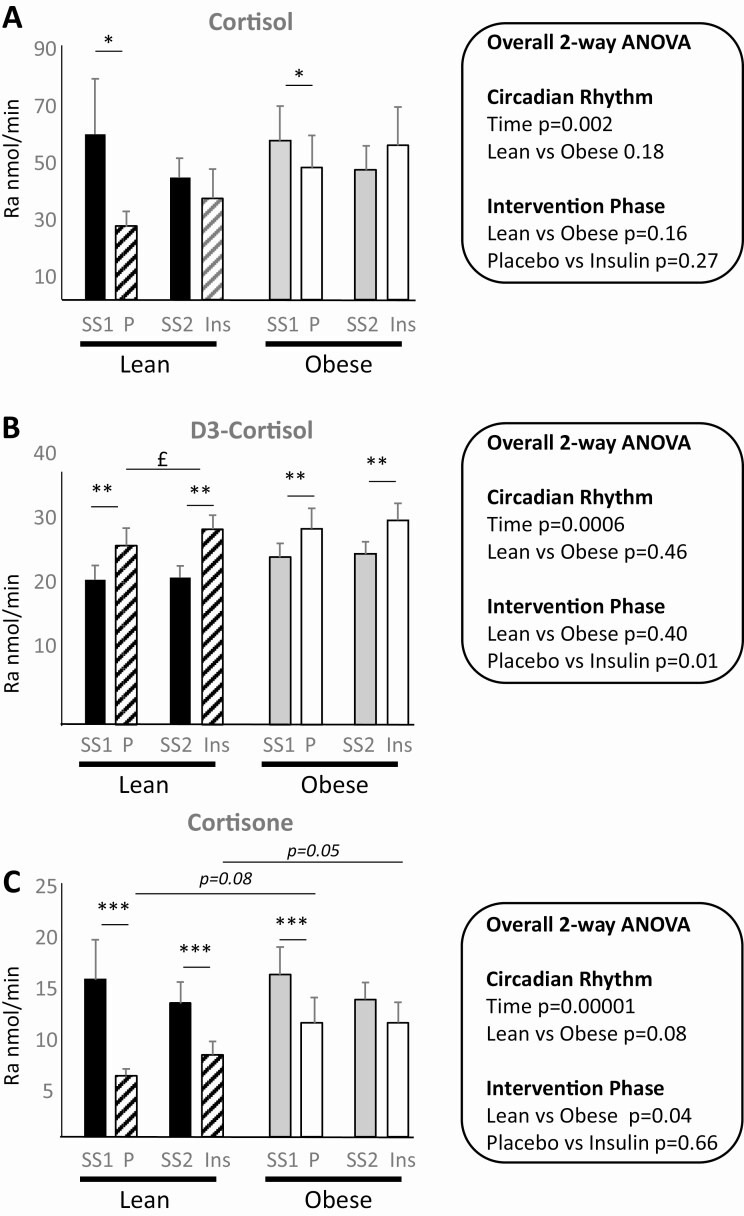
Whole-body rates of appearance of A, cortisol; B, D3-cortisol; and C, cortisone. Data show lean and obese participants studied on 2 occasions. In each case rates were quantified at steady-state periods and then during intervention, either under continued placebo infusion or alternatively insulin. Data are mean ± SEM, n = 10/group, except in lean and obese participants during placebo intervention, where n = 9. Two sets of analyses were performed, and overall analysis of variance (ANOVA) results are indicated in the figure inserts and post hoc tests are shown within the histograms. Rates of appearance were compared between lean and obese participants at steady state and then later in the day under prolonged placebo infusion using a repeated-measure ANOVA (testing effect of obesity and time of day). Secondly, during the intervention phase, the effect of insulin vs placebo was compared using a 2-way ANOVA (testing effect of obesity and insulin). Fisher post hoc tests were applied in both cases. *P less than .05; **P less than .01; and ***P less than .005 and £*P* less than .05, where * compares steady-state and intervention phases and £ compares intervention phases. Strong trends *P* less than .10 are indicated. SS1, steady-state preplacebo; SS2, steady-state preinsulin; P, placebo; Ins, insulin; Ra, rate of appearance.

At steady state (ie, before intervention), circulating concentrations of endogenous cortisol and cortisone declined in arterialized blood until the end of the steady-state period at 180 minutes (*P* < .05; Fig. 2A and 2B) and were not different between lean and obese participants. Concentrations of infused tracers increased until 120 minutes, when steady state was achieved (Fig. 2C-2E). At steady state, clearance of D2-cortisone was more rapid overall in obese participants, but clearance of D4-cortisol did not differ between groups (Table 2). The whole-body Ra of endogenous cortisol, D3-cortisol, and cortisone (a measure of 11β-dehydrogenase activity) did not differ between lean and obese participants (Fig. 3).

**Table 2.** Clearance of tracers and endogenous steroids in lean and obese participants at steady state and during a hyperinsulinemic clamp

					Clearance, L/min			
					Lean		Obese	
	Phase	Effect of obesity	Circadian effect	Effect of insulin	Placebo (n = 10, 9)	Insulin (n = 10, 10)	Placebo (n = 10, 9)	Insulin (n = 10, 10)
D4-cortisol	Steady state	NS			0.47 ± 0.06	0.45 ± 0.06	0.54 ± 0.07	0.56 ± 0.07
	Intervention (270-300 min)	NS	*P* = .07	NS	0.49 ± 0.07	0.47 ± 0.05	0.60 ± 0.09^a^	0.56 ± 0.06
D2-cortisone	Steady state	*P* < .01			0.48 ± 0.04	0.48 ± 0.03	0.59 ± 0.04	0.62 ± 0.04^*d*^
	Intervention (270-300 min)	*P* < .001	*P* < .001	NS	0.39 ± 0.03^*c*^	0.40 ± 0.01	0.50 ± 0.04^*a*,*b*^	0.55 ± 0.03^*b*^

Effect of obesity was assessed using steady-state data. Circadian changes were assessed comparing steady-state and placebo intervention data. Effects of hyperinsulinemia were assessed by comparing data within the intervention phases (placebo vs insulin). In all cases repeated-measures analyses of variance were used with Fisher least significant difference post hoc tests. Data are mean ± SEM, n (steady state, intervention).

Abbreviation: NS, not significant.

^*a*^and ^*b*^*P* less than .05 and ^*c*^and ^*d*^*P* less than .01, where ^*a*^refers to paired steady state vs placebo reflecting circadian change and ^*b*^refers to obese vs lean comparing data against paired (placebo and insulin) interventions.

#### Effect of hyperinsulinemia (vs prolonged placebo) intervention

During hyperinsulinemia (180-300 minutes), mean glucose was successfully maintained at physiological concentration (lean placebo 5.32 ± 0.17 vs lean insulin 4.90 ± 0.13 vs obese placebo 5.25 ± 0.14 vs obese insulin 4.92 ± 0.26 mmol/L). During placebo infusion circulating concentrations of cortisol continued to decline following natural circadian rhythm, but to a lesser extent in the presence of insulin (interaction between intervention and time *P* = .005 from 180 to 300 minutes; Fig. 2A). The effect of obesity on these findings was further explored using planned comparisons between the Ra using values averaged across steady state as comparator. The rate of whole-body cortisol generation from steady state decreased toward the end of the intervention phase both in lean and obese participants receiving placebo, but not following insulin infusion in either group (Fig. 3A). Conversely, during placebo infusion the rate of D3-cortisol generation (the specific measure of 11β-reductase activity) increased across the intervention, and this was further increased overall by insulin (Fig. 3B). A difference was not found between obese and lean groups, but the increase reached significance only the lean participants. The rate of cortisone generation declined during the placebo intervention, both in lean and obese individuals. This decline was still observed in lean participants following insulin but not in the obese men (Fig. 3C).

Clearance of D4-cortisol tended to increase across the day, but reached significance only in obese participants, whereas D2-cortisone clearance declined across the day both in the lean and obese groups (see Table 2), remaining higher in obese men. Insulin did not alter clearance of D4-cortisol or D2-cortisone.

### Reductase and dehydrogenase activities across adipose tissue

**Figure 4.**
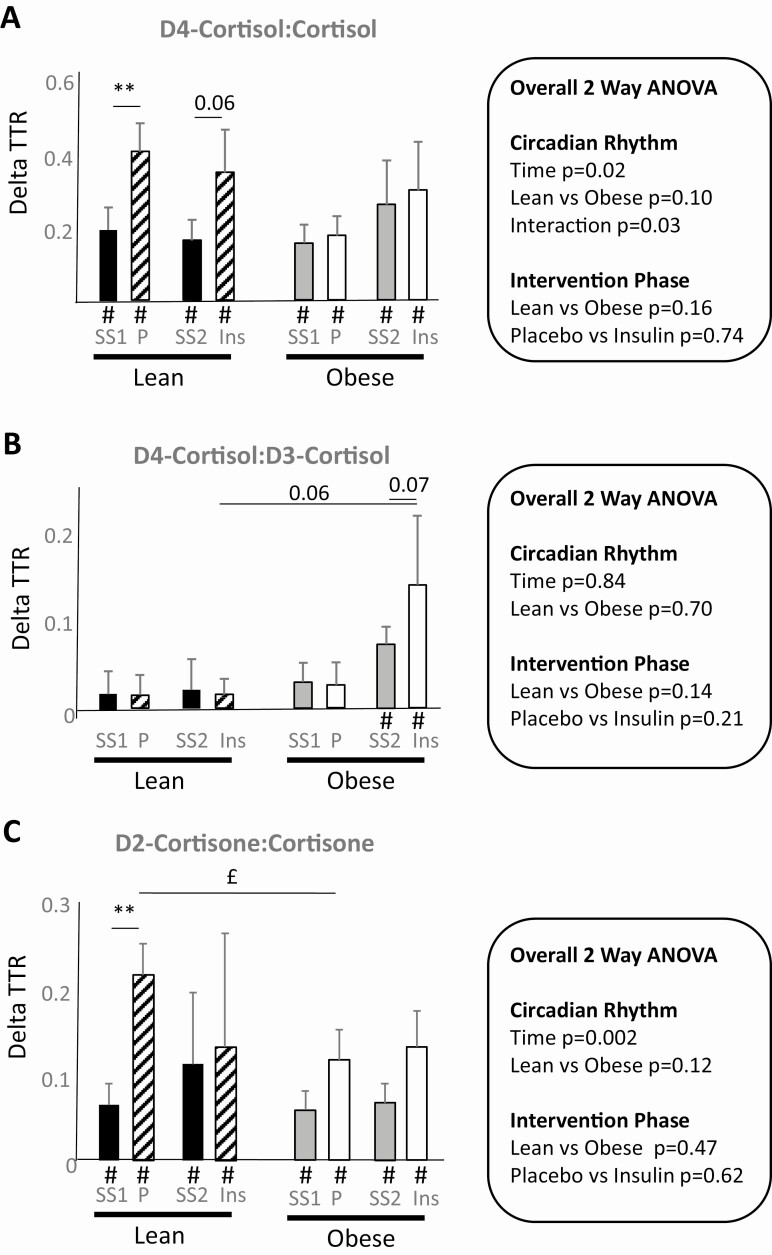
Change in tracer:tracee ratio (TTR) across adipose tissue A, D4-cortisol:cortisol; B, D4-cortisol:D3-cortisol; and C, D2-cortisone:cortisone. Data show lean and obese participants studied on 2 occasions. In each case ΔTTR was quantified at steady-state periods and then during intervention, either under continued placebo infusion or alternatively insulin. Data are mean ± SEM. Measurable appearance/extraction of tracee across adipose was assessed by dilution of tracer across adipose compared against zero by one-sided *t* test and indicated by #*P* less than .05. Two sets of analyses were performed, and overall analysis of variance (ANOVA) results are indicated in the figure inserts and post hoc tests are shown within the histograms. Rates of appearance were compared between lean and obese participants at steady state and then later in the day under prolonged placebo infusion using a repeated-measure ANOVA (testing effect of obesity and time of day). Secondly, during the intervention phase, the effect insulin vs placebo was compared using a 2-way ANOVA (testing effect of obesity and insulin). Fisher post hoc tests were applied in both cases. * and £* P* less than .05 and **P less than .01, where * compares steady-state and intervention phases and £ compares intervention phases. Strong trends *P* less than .10 are indicated. SS1, steady-state preplacebo; SS2, steady-state preinsulin; P, placebo; Ins, insulin; Ra, rate of appearance. n = 9 except in the lean group during steady state 2 (preinsulin) and both lean and obese participants during insulin infusion.

Generation of cortisol and cortisone were detected reliably by tracer dilution across adipose tissue at steady state and during the intervention period both in lean and obese individuals (Fig. 4A and 4C). In lean men only, dilution of tracer both by cortisol and cortisone increased across the day but was unaffected by insulin (see Fig. 4C). Dilution of cortisone tracer across adipose likewise was detected at all time points and did not differ between lean and obese participants, with dilution increasing generally across the day (see Fig. 4C). Generation of D3-cortisol (Fig. 4B) across adipose was detected only in obese men and dilution of tracer was more marked following insulin infusion.

When arteriovenous Ra were calculated by adjusting tracer dilution for blood flow (Table 3), differences in the production of cortisol and cortisone did not differ between lean and obese participants at steady state across the day or in response to insulin. D3-cortisol generation was higher in obese than lean men during insulin infusion.

**Table 3.** Generation of cortisol, D3-cortisol, and cortisone across adipose tissue and skeletal muscle in lean and obese participants before and after insulin infusion

	Summary ANOVA *P*			Steady-state		Placebo intervention		Steady-state		Insulin intervention	
				120-180 min (preplacebo)		270-300 min		120-180 min (preinsulin)		270-300 min	
	Obesity	Circadian	Insulin	Lean	Obese	Lean	Obese	Lean	Obese	Lean	Obese
	at SS	Circ*Ob	Ins*Ob								
Adipose Ra steroids, pmol/100 g tissue											
No.				9	9	9	9	8	9	8	8
Blood flow^				1.92 ± 0.25	1.51 ± 0.47	1.68 ± 0.12	1.48 ± 0.47	2.10 ± 0.27	1.34 ± 0.31	1.62 ± 0.23	1.62 ± 0.43
Cortisol	.54	.11	.93	44.89 ± 19.51	15.51 ± 4.33	57.87 ± 33.51	59.33 ± 40.48	48.38 ± 27.48	37.05 ± 17.16	44.35 ± 32.13	67.53 ± 40.53
		.76	.72								
D3-Cortisol	.20	.72	.12	3.23 ± 3.52	5.22 ± 2.03	4.08 ± 3.83	3.84 ± 2.86	1.12 ± 1.32	11.68 ± 3.63	3.53 ± 3.72	18.99 ± 9.62$
		.63	.09								
Cortisone	.44	.51	.33	5.46 ± 2.01	2.72 ± 0.59	4.65 ± 1.19	1.41 ± 2.57	20.79 ± 20.02	4.38 ± 1.68	18.29 ± 17.52	4.29 ± 4.69
		.98	.49								
Skeletal muscle Ra steroids, pmol/100 g tissue											
No.				10	10	9	9	10	10	10	10
Blood flow^*b*^				2.67 ± 0.42	3.60 ± 0.18	3.01 ± 0.54	3.91 ± 0.45	2.75 ± 0.38	3.39 ± 0.42	2.72 ± 0.47	4.06 ± 0.67
Cortisol	.05	.84	.38	20.44 ± 9.43	–5.15 ± 8.39	22.58 ± 13.54	–1.62 ± 4.03	18.62 ± 7.10	6.07 ± 4.20	4.50 ± 10.32	39.94 ± 20.51^*a*^
		.82	.04								
D3-Cortisol	.20	.85	.13	5.41 ± 3.03	14.40 ± 9.9	12.59 ± 4.01	8.80 ± 5.23#	–9.64 ± 12.19	1.52 ± 2.95	–7.68 ± 15.69	1.15 ± 1.90
		.15	.48								
Cortisone	.84	.12	.98	7.76 ± 1.59	7.42 ± 5.75	4.50 ± 1.42	3.21 ± 2.59	9.75 ± 1.83	11.17 ± 3.68	2.55 ± 0.90 $	5.10 ± 2.39
		.84	.15								

Effect of obesity was assessed using steady-state data. Circadian changes were assessed comparing steady-state and placebo intervention data. Effects of hyperinsulinemia were assessed by comparing data within the intervention phases (placebo vs insulin). In all cases repeated-measure ANOVAs were used with Fisher least significant difference post hoc tests. Data are mean ± SEM.

Abbreviations: ANOVA, analysis of variance; Ra, rate of appearance; SS, steady-state.

^*a*^Placebo vs insulin during the intervention phase.

^*b*^Blood flow expressed as mL/min/100 g tissue.

### Reductase and dehydrogenase activities across skeletal muscle

**Figure 5.**
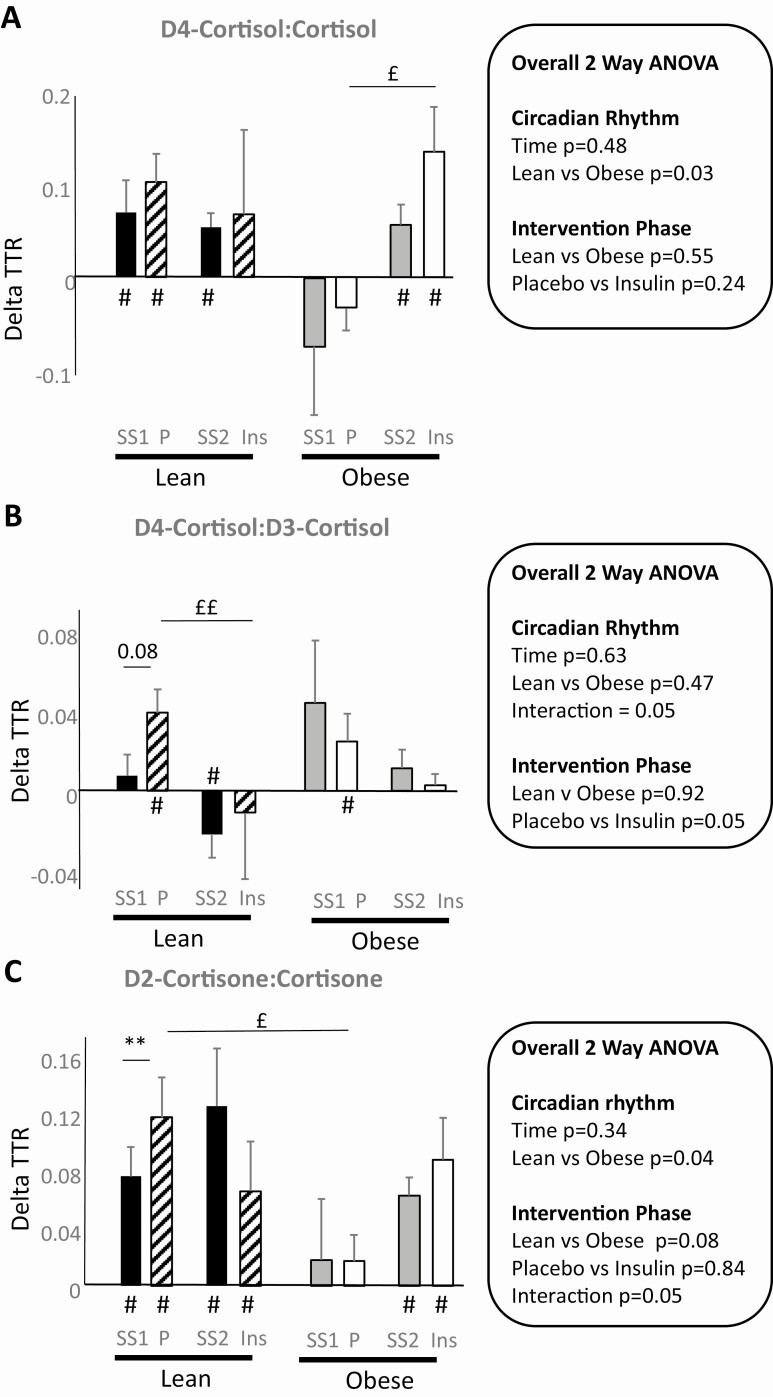
Change in tracer:tracee ratio (TTR) across skeletal muscle A, D4-cortisol:cortisol; B, D4-cortisol:D3-cortisol; and C, D2-cortisone:cortisone. Data show lean and obese participants studied on 2 occasions. In each case ΔTTR was quantified at steady-state periods and then during intervention, either under continued placebo infusion or alternatively insulin. Data are mean ± SEM. Measurable appearance/extraction of tracee across adipose was assessed by dilution of tracer across skeletal muscle compared against zero by one-sided *t* test and indicated by #*P* less than .05. Two sets of analyses were performed, and overall analysis of variance (ANOVA) results are indicated in the figure inserts and post hoc tests are shown within the histograms. Rates of appearance were compared between lean and obese participants at steady state and then later in the day under prolonged placebo infusion using a repeated-measure ANOVA (testing effect of obesity and time of day). Secondly, during the intervention phase, the effect of insulin vs placebo was compared using a 2-way ANOVA (testing effect of obesity and insulin). Fisher post hoc tests were applied in both cases. * and £ *P* less than .05 and ** and ££*P* less than .01, where * compares steady-state and intervention phases and £ compares intervention phases. Strong trends *P* less than .10 are indicated. SS1, steady-state preplacebo; SS2, steady-state preinsulin; P, placebo; Ins, insulin; Ra, rate of appearance. n = 10/group except during the placebo intervention, where n = 9 both in the lean and obese groups.

In lean participants, dilution of cortisol and cortisone tracer was detected at steady state (Fig. 5A and 5C). Dilution of cortisol tracer across skeletal muscle was unchanged after prolonged infusion of placebo or insulin. Dilution of cortisone increased on placebo infusion in lean subjects but not with insulin. Dilution of tracers of cortisol and cortisone at steady state was not reliably detected in obese subjects, but was measured following insulin infusion. Dilution of tracer by D3-cortisol was not detected at steady state in either lean or obese participants and was detected only following the placebo intervention phase both in lean and obese participants and not after insulin.

Adjusting for blood flow, greater cortisol production was detected in lean men compared to obese at steady state. Obese participants generated more cortisol during insulin infusion compared with placebo infusion and also lean men receiving insulin. In the case of cortisone, insulin reduced generation in lean participants only. No differences were observed in D3-cortisol production between treatments.

### Glucocorticoid-regulated transcripts in adipose

The abundance of transcripts of *HSD11B1*, *HSD11B2*, *PER1*, and *NR3C1* were unaffected by obesity or insulin (Table 4).

**Table 4.** Transcripts quantified by quantitative polymerase chain reaction in adipose biopsies before and after intervention (placebo or insulin infusion) in lean and obese men

	Lean		Obese	
	Placebo	Postinsulin	Placebo	Postinsulin
*PER1*	1.02 ± 0.35	1.14 ± 0.17	0.71 ± 0.23	0.62 ± 0.17
*NR3C1*	0.82 ± 0.10	0.92 ± 0.15	0.62 ± 0.03	0.67 ± 0.04
*HSD11B1*	0.61 ± 0.14	0.54 ± 0.04	0.69 ± 0.14	0.89 ± 0.29
*HSD11B2*	0.75 ± 0.27	0.87 ± 0.14	0.61 ± 0.16	0.88 ± 0.33

Data are mean ± SEM of transcript abundance corrected for that of the housekeeping gene. Lean, n = 7; obese, n = 6 per group.

## Discussion

These data confirm that, as previously reported (16), there is recycling between cortisol and cortisone in human subcutaneous adipose tissue and skeletal muscle in vivo. In the absence of substantial amounts of 11βHSD2 in these 2 tissues, we attribute both the 11β-reductase and 11β-dehydrogenase catalytic reactions to 11βHSD1; this contrasts with the whole-body measurements in which liver 11βHSD1 contributes the vast majority of 11β-reductase activity (4, 34-36), whereas kidney 11βHSD2 accounts for most 11β-dehydrogenase activity. In nondiabetic, obese men, there was no substantial shift in the equilibrium between cortisol and cortisone either in the systemic circulation or in adipose tissue or skeletal muscle when fasting compared with lean participants, although 11β-reductase activity, measured by D3-cortisol generation, was more readily detected in obese men across adipose under hyperinsulinemic conditions. During hyperinsulinemia, 11β-dehydrogenase activity in skeletal muscle was not downregulated in response to insulin in obese men, unlike lean men.

Early assessments of hepatic and adipose 11βHSD1 suggested tissue-specific dysregulation in obesity, with reduced first-pass metabolism of oral cortisone to cortisol across the splanchnic bed in vivo and increased enzyme activity measured ex vivo in biopsies (26, 37). More recent use of deuterated steroid tracers has allowed direct measurement of 11βHSD1 activity both in the whole body and across tissue beds (25), although of note, dehydrogenase activity measured by cortisone dilution of D2-cortisone does not distinguish between the contributions of 11βHSD1 and 11βHSD2. One limitation of the technique is that cortisol, D3-cortisol, and cortisone production rates are all affected by substrate concentration; in adipose tissue there is relatively slow accumulation of tracer (38) and hence measured production rates may diverge from whole-body kinetics, even during prolonged infusion over 6 hours. In some previous studies (16), higher tracer concentrations systemically were used (to overcome analytical limitations), which may have facilitated detection of enzymatic activity in tissues, although lower tracer levels are desirable in these studies. Temporal changes are exacerbated by circadian variation in hypothalamic-pituitary-adrenal axis activity and hence cortisol secretion. These factors are likely to have contributed to some observations in this study, for example, the change in Ra of cortisone and the slower decline in cortisol production in obesity seen here, the latter mirroring previous observations of flattened diurnal rhythm in plasma cortisol (39). As a result of using sufficient tracer to be reliably detected in blood, the tracer may have suppressed the hypothalamic-pituitary-adrenal axis and this will have contributed to the suppression of cortisol production throughout the day. However, it is interesting to note that hyperinsulinemia further flattened the diurnal rhythm in cortisol and cortisone production, without any influence on tracer clearance and hence tracer steroid concentrations. This finding could be explained by a concomitant increase in cortisol regeneration by insulin, reflecting whole-body 11β-reductase activity, in lean but not in obese men, suggesting the effect of insulin may influence alternative metabolic routes or also act directly on cortisol production via the adrenal gland.

Nonetheless, in keeping with previous studies, the rates of whole-body appearance of cortisol or D3-cortisol in the “metabolically healthy” obese men studied here were not different from lean individuals (3, 40, 41); whole-body D3-cortisol generation has previously been shown to increase only when obesity is accompanied by type 2 diabetes (2, 32). Insulin sensitivity assessed by M values was shown to be comparable between the obese and lean groups, and indeed in the healthy range (42). Other differences between the groups studied here and those previously published might also be relevant, for example, the wide age range of both the lean and obese groups. However, other studies have shown similar whole-body 11βHSD1 activity across a variety of age ranges in healthy lean and obese individuals, as well as in obese diabetic individuals (2, 32), suggesting that age is not the primary factor influencing whole-body enzyme activity.

For adipose tissue, cortisol and cortisone generation were not different in obese vs lean men but D3-cortisol generation was detected across adipose only in obese participants. These findings are consistent with previous reports of increased regeneration of cortisol within adipose tissue in obesity, for example, using local steroid infusions with microdialysis (3, 17) or arteriovenous sampling (17), and with one previous study showing no change in dehydrogenase activity in adipose in vivo (41). We conclude that in the fasted state, the weight of evidence supports an increase in reductase but no change in dehydrogenase activity in adipose tissue in obesity.

Substantial literature implicates insulin in the regulation of 11βHSD1, and it has been inferred that altered insulin signaling underlies dysregulation of 11βHSD1 in obesity (43). One potential mediator of insulin’s effects is the hexose-6-phosphate pathway, which determines NADPH availability and hence the reductase/dehydrogenase equilibrium of 11βHSD1 (44). In a previous study, the rate of generation of D3-cortisol was increased by insulin infusion in the whole body in lean participants (3, 18). Other reports show reductase but not dehydrogenase activity was transiently increased in adipose tissue, measured by radiotracer and microdialysis (18), although in contrast adipose reductase activity measured by microdialysis declined during insulin infusion (3). Insulin is also the likely mediator of increases in whole-body D3-cortisol production in response to feeding (45), most particularly in response to meals that stimulate insulin secretion (carbohydrate more so but protein to some extent) and this may contribute to increases in circulating cortisol postprandially. Few studies have investigated glucocorticoid kinetics across skeletal muscle in vivo. Using D4-cortisol to measure 11β-reductase activity, Basu et al did not detect cortisol generation across leg skeletal muscle in a cohort of obese individuals either at steady state or during a hyperinsulinemic euglycemic clamp (36), whereas Hughes et al were able to quantify cortisol and D3-cortisol generation in forearm skeletal muscle (16).

The metabolic consequences of such findings with increased local regeneration of cortisol in subcutaneous adipose tissue are highlighted from the results of previous studies and the consequences of Cushing syndrome for adipose tissue. Enhanced glucocorticoid receptor activation by local upregulation of cortisol reactivation may further enhance 11βHSD1 expression and activity (46). The synergistic effects of elevated cellular cortisol and insulin, for example, on lipoprotein lipase activity, result in insulin resistance and impaired glucose control with elevated circulating free fatty acids (47).

In the present study, hyperinsulinemia attenuated the decline in cortisol production with time both in lean and obese men and exaggerated the increase in D3-cortisol generation, consistent with previously observed induction of whole-body 11βHSD1 and most likely reflecting changes in hepatic enzyme activity. Within adipose tissue and skeletal muscle, effects of insulin were variable. Dehydrogenase activity in skeletal muscle decreased in response to insulin in lean participants only. In adipose there was an increase D3-cortisol generation in response to insulin in obese participants only. Overall, this suggests that hyperinsulinemia contributes to enhanced cortisol regeneration rather than cortisol inactivation in adipose tissue and this effect is exaggerated in metabolically healthy obese men. Changes in glucocorticoid-regulated transcripts were not observed over the short period of study in adipose and it remains uncertain whether the magnitude of enzyme activity in skeletal muscle is sufficient to affect glucocorticoid signaling. It is unknown whether such findings would translate in the setting of insulin resistance in individuals with type 2 diabetes mellitus.

Tracer studies also provide the gold standard for measuring metabolic clearance. Unexpectedly, the clearance of the D4-cortisol tracer did not differ between lean and obese participants. Previous studies have documented increased cortisol clearance in obesity (48, 49) and a trend toward an increase has been reported with D4-cortisol infusion (3, 44). However, we made the novel observation that cortisone clearance is more rapid in obesity; this may reflect an increase in cortisone metabolism by 5β-reductase and/or 11βHSD1; the latter may be supported by a trend toward an increase in D3-cortisol generation. Clearance of D4-cortisol tracers increased and that of D3-cortisone decreased across the day, but this was unaffected by insulin, further suggesting insulin may act directly to influence steroid regeneration or steroidogenesis.

Limitations of the study include some incomplete data sets due to difficulty in sampling across abdominal wall subcutaneous adipose tissue, particularly after prolonged cannulation. This may have masked differences between groups at other times. Measures of blood flow became more variable at the later time points, as is common when participants become uncomfortable after prolonged immobilization, so data have been presented both on the basis of changes in tracer dilution as well as absolute rates of production. In smaller experimental medicine studies such as these, variability in potential measured or unmeasured confounders such as age and insulin sensitivity cannot be adjusted for statistically and may influence the results when comparing groups, although less so in the paired analyses of the effects of insulin.

In conclusion we have shown that cortisol and cortisone recycling occurs both in adipose tissue and skeletal muscle in humans in vivo. During hyperinsulinemia, the equilibrium shifts in favor of active cortisol, particularly in adipose tissue. In obesity, this phenomenon may be exaggerated after meals. However, we did not demonstrate that dehydrogenase activity in skeletal muscle or adipose tissue is substantially upregulated in obesity or by insulin. This suggests that the modest metabolic benefits from 11βHSD1 inhibition (11-13) are unlikely to be improved on dramatically by compounds that inhibit reductase and not dehydrogenase activity.

## Data Availability

Some or all data sets generated during and/or analyzed during the present study are not publicly available but are available from the corresponding author on reasonable request.

## Abbreviations

11βHSD1: 11β-hydroxysteroid dehydrogenase 1
11βHSD2: 11β-hydroxysteroid dehydrogenase type 2
ANOVA: analysis of variance
NADP: nicotinamide adenine dinucleotide phosphate
Ra: rates of appearance

## Additional Information

***Disclosures:*** B.R.W. is an inventor on patents for 11β-HSD1 inhibitors owned by University of Edinburgh and licensed to Actinogen Medical. B.R.W. and R.A. are consultants for Actinogen Medical. The other authors have nothing to disclose.
